# A Multi-Scale Approach to Investigate Adhesion Properties of *Pseudomonas aeruginosa* PAO1 to *Geotrichum candidum* LG-8, a Potential Probiotic Yeast

**DOI:** 10.3390/foods9070912

**Published:** 2020-07-11

**Authors:** Ling Meng, Siduo Zhou, Xiao Xu, Dian Li, Yanfei Lin, Fangxin Lyu, Mingsheng Dong

**Affiliations:** College of Food Science and Technology, Nanjing Agricultural University, 1 Weigang Road, Nanjing 210095, China; chinaling0@163.com (L.M.); zhousiduo@126.com (S.Z.); xiaolexu@126.com (X.X.); lidianlucky@163.com (D.L.); 2017108010@njau.edu.cn (Y.L.); cindylfx1997@outlook.com (F.L.)

**Keywords:** adhesion, *Geotrichum candidum*, *Pseudomonas aeruginosa*, microscopy, polysaccharide

## Abstract

This study investigated properties of *Pseudomonas aeruginosa* PAO1 adhesion to *Geotrichum candidum* LG-8 cells in variable pH and salt conditions. The primary mechanism was revealed by multi-scale microscopy technics. The adhesion of PAO1 to the living fungus occurred within 1 h and was limited at concentrations of bile salts higher than 0.5%. The adhesion efficiency gradually increased to 58.1% with the pH increasing from 2.0 to 7.0 and then decreased to 48.2% at pH 9.0. However, the dead LG-8 has an advantage over the living ones to adhere PAO1 in same pH and bile salt conditions. Optical microscopy showed that both unsterilized and sterilized *G. candidum* LG-8 cells removed approximately one hundred fold bacteria in 4 h. Laser scanning confocal microscopy (LSCM) analysis indicated that polysaccharides of the fungus contributed to adhesion. Scanning electron microscopy (SEM) analysis proved that syrup-like EPS (extracellular polymeric substances) of LG-8 coating PAO1 was in part a mechanism. Atomic force microscopy (AFM) showed roughness of the LG-8 surface changed in the adhesion process. Furthermore, a pedestal-like structure of bacteria was observed by transmission electron microscopy (TEM) analysis, indicating that the bacteria were also actively involved in the adhesion process. *G. candidum* LG-8 is a potential candidate for the control of *P. aeruginosa* PAO1 in the food industry and immunodeficiency patients.

## 1. Introduction

*Pseudomonas aeruginosa* is a common foodborne and waterborne pathogenic bacterium. It can cause spoilage of food by consuming the nutrients in food and/or producing certain molecules [1]. According to the World Health Organization (WHO), 70% to 80% of human diseases are caused by unclean drinking water. With the improvement of living quality, water quality has attracted consumers’ attention. However, reports of *P. aeruginosa* detected in packaged drinking water are increasing, indicating a direct threat to consumer health [2,3]. In addition, *P. aeruginosa* is a common opportunistic human pathogen that rarely poses a threat to healthy individuals. However, it poses a major threat to immunocompromised individuals and cystic fibrosis patients with underlying conditions [4]. Furthermore, *P. aeruginosa* can thrive and reproduce easily, showing strong resistance to antibiotics and a tendency to generate acquired resistance [5]. The threat can lead to high mortality and morbidity [6]. Furthermore, as a pathogen of the family *Pseudomonadaceae*, *P. aeruginosa* is able to thrive and reproduce easily in a wide range of environmental conditions, showing strong resistance to chemical and physical factors such as disinfectants and ultraviolet radiation [7,8]. Therefore, the various adaptive responses and genetic mutations result in *P. aeruginosa* having strong resistance to antibiotics and a tendency to generate acquired resistance [5,9]. To treat *P. aeruginosa* contamination, novel antimicrobial approaches are necessary.

Although the main ways to inactivate pathogenic bacteria are physical and chemical methods [10,11], the use of microorganisms such as probiotics has been proposed as a potential strategy to treat and prevent gastrointestinal disorders [12]. However, few eukaryotic probiotics have been well studied. Although only *Saccharomyces boulardii* has been commercialized worldwide, investigations on the application of other yeasts and yeast-like species or genera have been carried out based on clinical trials and in vitro assays [13,14]. Probiotics are available from a variety of sources, reducing costs and avoiding risks associated with the extraction process.

Cell adhesion mediated by relevant molecules is one of the most basic life phenomena and is also the basis of inflammation, infection, immunity, etc. [15,16]. Disruption of cell adhesion has become an important strategy for controlling diseases. A great advantage of fungi is their ability as eukaryotic probiotics to express some therapeutic proteins in their active conformations [13,17]. Furthermore, probiotics provide a variety of sources, reduce costs, and avoid other risks associated with the extraction process.

A sizable proportion of studies on the phenomenon and mechanism of pathogenic contamination and infection were mainly carried out by physiological and biochemical methods. Nonetheless, a single instrument can no longer meet the needs of scientific research. Thus, combined analysis with diverse instruments is being promoted. Microscopy instruments have been widely used in combined analysis. For example, multiple antibacterial investigations were performed via electron microscopy and flow cytometry [18,19]. Moreover, some reports assessed bacterial viability in organic solvents at the cellular level via atomic force microscopy (AFM) and laser scanning confocal microscopy (LSCM) [20]. 

In our present work, the efficiency of *P. aeruginosa* PAO1 adhering to the surface of *Geotrichum candidum* LG-8 cells was evaluated under various conditions (pH, time, fungal viability, and bile salt). The mechanism of the fungus action was in part investigated by LSCM, scanning electron microscopy (SEM), transmission electron microscopy (TEM), and AFM. Adhesion of the pathogenic bacteria to *G. candidum* LG-8 (isolated from kefir) explains in part the protective effect of probiotics.

## 2. Material and Methods

### 2.1. Strains and Culture Conditions

Both the *P. aeruginosa* PAO1 strain (a pathogenic bacterium isolated from a wound) and *G. candidum* LG-8 strain (MK640636, a yeast-like strain isolated from kefir) were obtained from the Food Microbiology Laboratory of Nanjing Agricultural University (Nanjing, China). *P. aeruginosa* PAO1 was subcultured three times in LB (1% Tryptone, 0.5% Yeast extract, 0.5% NaCl) broth for 22 h at 37 °C and 180 r/min. *G. candidum* LG-8 was revived on solid YPD medium (2% glucose, 2% peptone, 1% yeast extract, 1.5% agar) at 30 °C for 24 h, and subsequently incubated twice in YPD broth at 30 °C and 180 r/min for 24 h. The two strains were subcultured twice in broth prior to experimental usage. Finally, the *P. aeruginosa* PAO1 and *G. candidum* LG-8 cells above were centrifuged at 2611× *g* for 3 min and 940× *g* for 3 min, respectively, and then stored in sterilized water after washing three times by sterile saline.

### 2.2. P. aeruginosa PAO1 Adhesion Assays

To evaluate the ability of *G. candidum* LG-8 to remove *P. aeruginosa* PAO1, studies were performed as follows: unsterilized (living) LG-8 cells and *P. aeruginosa* PAO1 cells were diluted with sterile saline to concentrations of 10^7^ CFU/mL and 10^8^ CFU/mL, respectively. Then, 1.2 mL of *G. candidum* LG-8 was mixed with 0.3 mL of *P. aeruginosa* PAO1. The mixed cells were incubated at 37 °C for 4 h. Afterwards, 100 μL of suspension was then immediately spread on LB solid medium and cultured at 37 °C for 24 h. The control group contained only *P. aeruginosa* PAO1 cells.

Sterilized (dead) LG-8 cells were sterilized at 121 °C for 20 min. Then, the cells were diluted and the adhesion assays were performed according to the abovementioned methods in this section.

### 2.3. Evaluation of Factors Influencing P. aeruginosa PAO1 Adhesion

Adhesion between *P. aeruginosa* PAO1 and *G. candidum* LG-8 may be affected by the environment. We therefore investigated adhesion efficiency under variable conditions (time, fungal viability, pH, and the presence of oxgall bile salts).

Effects of time: To visually determine whether the *P. aeruginosa* PAO1 were bound, a drop of the sample was dyed based on the Gram staining method and visualized by optical microscopy at intervals of 0 min, 1, 2, 3, 4, 6, 12, and 24 h. The remaining assays were performed according to Section 2.2.

Effects of LG-8 viability: LG-8 cells sterilized by moist heat at 121 °C for 15 min were taken as the sterilized group, while cells untreated with moist heat were taken as the unsterilized group. Then, the cells were taken to adhesion assay described in Section 2.2 “*P. aeruginosa* PAO1 adhesion assays”.

Effects of pH: to determine the adhesion efficiency of *P. aeruginosa* PAO1 by *G. candidum* LG-8 under different pH conditions, the pH of the solution containing the cells was adjusted to 2.0–9.0 with 1 M HCl or 1 M NaOH. Moreover, the influence of oxgall bile salts on the bacterial adhesion efficiency was studied. Both bacterial and fungal cells were diluted in the same saline solution containing 0.1%, 0.2%, 0.3%, 0.5%, or 1% (*w/v*) oxgall bile salts. The remaining assays were performed according to methods described in Section 2.2 “*P. aeruginosa* PAO1 adhesion assays”.

Our preliminary experiments showed that sterilized *P. aeruginosa* PAO1 still adhered to *G. candidum* LG-8. Therefore, considering the impact of sterilized *P. aeruginosa* PAO1 on the evaluation of the bacteria adhesion to *G. candidum* LG-8, adhesion efficiency was monitored by optical density at 600 nm with an ultraviolet spectrophotometer (UV-2802PC, UNICO, Shanghai, China) and calculated according to the methods demonstrated by Vandevoorde et al. [21]. Briefly, after incubation in variable pH solutions and the solutions at different concentrations of oxgall bile salts in this section, bacterial cells and fungal cells were harvested by centrifuging at 940× *g* for 3 min. Then, those cells were resuspended in their initial incubation solutions. The optical densities were finally measured at 600 nm. The adhesion efficiency was calculated by Equation (1) as follows

(1) $$\mathrm{Adhesion}\;\mathrm{efficiency}\;\left(\%\right)=\frac{\left(\mathrm{OD}.A+\mathrm{OD}.B\right)\;-\;2\;\times \;\mathrm{OD}.\mathrm{AB}}{\mathrm{OD}.A+\mathrm{OD}.B}\times 100$$

where OD.AB: optical density of mixed *G. candidum* LG-8 and *P. aeruginosa* PAO1. OD.A: optical density of *G. candidum* LG-8. OD.B: optical density of *P. aeruginosa* PAO1. 

### 2.4. Measurement via Optical Microscopy

After evaluation about effects of LG-8 viability on *P. aeruginosa* adhesion in Section 2.3, *G. candidum* LG-8 cells treated with *P. aeruginosa* cells were dyed according to the Gram staining method and observed by optical microscopy. *G. candidum* LG-8 untreated with *P. aeruginosa* was the control group.

### 2.5. Measurement via LSCM 

The fungal before or after bacterial-exposure were pretreated based on methods reported by Ding et al. [22]. Then, 200 μL of the cell suspension was mixed with 300 μL of fluoresce in fluorescein isothiocyanate labeled Concanavalin A (FITC-ConA) for 30 min at 4 °C in darkness. Excess cells and dye were removed by PBS (0.1 M, pH 7.4). Then, propidium iodide (PI) was added to the stained samples for 15 min at 4 °C in darkness. A drop (10 μL) of the sample was placed on a glass slide. The excitation wavelength and emission wavelength of FITC-ConA were 480 and 505/525 nm, respectively. PI was excited at 540 nm, while its emission wavelength was 560/660 nm. The detection parameters were adjusted to capture images. The areas associated with dead, compromised, and viable cells were visualized by LSCM (TCS SP8 STED 3×, Leica Microsystems Inc., Wetzlar, Germany; Ultra VIEW VoX, PerkinElmer, USA).

### 2.6. Measurement via SEM

The fungal cells with and without bacterial cells were fixed in 2.5% glutaraldehyde at 4 °C for 12 h. After being washed three times in PBS (0.1 M, pH 7.4), the cells were dehydrated with increasing alcohol concentrations (50%, 70%, 80%, 90%, and 100%). Isoamyl acetate (100%) was then used for the final dehydration treatment. A critical point driver with CO_2_ was used to dry the sample. After being placed on coverslips and sputtered with gold particles, samples were visualized by SEM (S-3000N, Hitachi Ltd., Tokyo, Japan).

### 2.7. Measurement via TEM

The pretreatment processes for TEM analysis were mostly consistent with methods described by Chen et al. [22] The first step was to fix the cells with glutaraldehyde (2.5%, *v/v*) for 12 h at 4 °C and 0.5 mL osmium tetroxide (1%) for 2 h. Then, 0.1 M sodium phosphate buffer (pH 7.4) was used to remove excess fixatives. A series of ethanol solutions (70%, 80%, 90%, and 100%) were then applied to dehydrate the samples for 10 min. The samples were sequentially immersed in propylene oxide for 15 min, a mixture of propylene oxide: Epon (1:1, *v/v*) for 1 h and a mixture of propylene oxide: Epon (1:3, *v/v*) for 2 h. Then, the samples were incubated in pure Epon at 37 °C for 6 h and at 60 °C for 12 h. The third step was to cut the resin blocks into ultrathin sections (70 nm) by an ultramicrotome device (EM UC7, Leica Microsystems Inc., Wetzlar, Germany). The ultrathin sections were stained with uranyl acetate for 20 min, and then washed thrice by pure water. Then, the sections were put in lead citrate for 10 min, mounted on copper TEM grids, and observed using a TEM instrument (JEM2100, JEOL Ltd., Tokyo, Japan).

### 2.8. Measurement via AFM

Cells were dropped onto a mica plate and immobilized by drying at room temperature. A Bruker Dimension Icon device (Dimension Icon, Bruker, Germany) was then used to determine the morphology of *G. candidum* LG-8 at room temperature with a relative humidity of 25–35%. Optimal scanning parameters were used to avoid cell damage or tip contamination. Thus, measurement was performed with an RTESP probe (RTESP-300, Bruker, CA, USA) in noncontact mode. The morphology, surface roughness (determined by a height sensor), and three-dimensional images provided information regarding the cell surface. The average roughness (Ra) was analyzed by AFM (Dimension Icon, Bruker, Karlsruhe, Germany) in ScanAsyst mode.

### 2.9. Statistical Analysis

The results are expressed as the means of at least three independent experiments. The data obtained were analyzed with one-way analysis of variance (ANOVA) through a Statistical Package for the Social Sciences (SPSS) software 20. Means were compared using Duncan’s honestly significant difference test (HSD, *p* < 0.05).

## 3. Results

### 3.1. Effect of Time and LG-8 Viability on P. aeruginosa PAO1 Adhesion

Visualization results at intervals of 0 min, 1, 2, 3, 4, 6, 12, and 24 h showed that adhesion of *P. aeruginosa* PAO1 to both sterilized (living) and unsterilized (dead) LG-8 clearly occurred within 1 h. However, adhesion also occurred among LG-8 cells, leading to reduce the surface area of LG-8 binding bacterial cells. Therefore, adhesion assays were performed within 4 h. The influence of LG-8 viability on *P. aeruginosa* PAO1 trapping was then studied. The abundances of *P. aeruginosa* PAO1 after treatment with unsterilized and sterilized LG-8 (Table 1) for 4 h were 4.96 and 5.13 log CFU/mL, respectively. Both sterilized and unsterilized LG-8 cells could still reduce the *P. aeruginosa* abundance by approximately one hundred-fold (from 7.01 to 5.13 and 4.96 log CFU/mL, respectively, Table 1) at 4 h. The results showed that *P. aeruginosa* PAO1 could adhere to both unsterilized and unsterilized LG-8. Additionally, the fungal LG-8 cells exhibited similar adhesion efficiency towards the bacterium.

### 3.2. Effect of pH on P. aeruginosa PAO1 Adhesion

**Figure 1:**
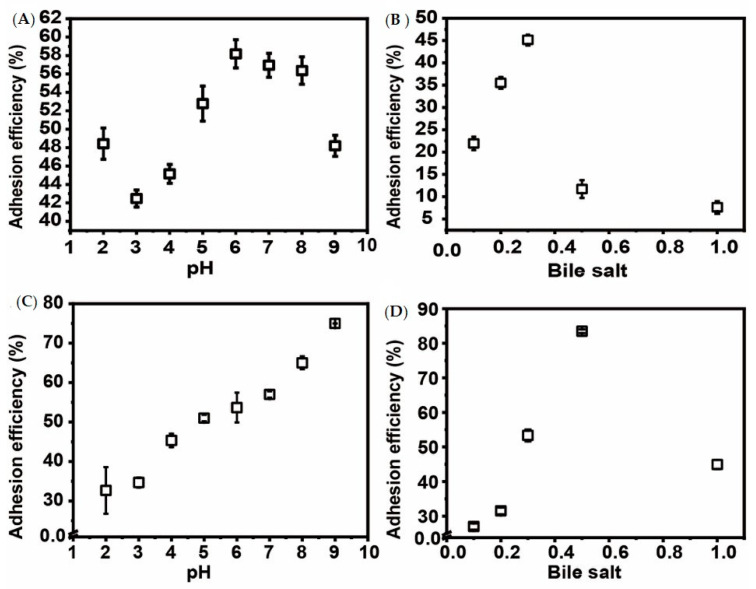
Effective factors on efficiency of *P. aeruginosa* PAO1 adhesion to living (**A**,**B**) or sterilized (**C**,**D**) *G. candidum* LG-8. (**A**,**C**): Factor, pH (2.0, 3.0, 4.0, 5.0, 6.0, 7.0, 8.0, and 9.0); (**B**,**D**): Factor, oxgall bile salts (0.1%, 0.2%, 0.3%, 0.5%, and 1%).

The gastrointestinal pH is variable. It is valuable to remove *P. aeruginosa* in a wide range of pH. Thus, the impact of pH on adhesion of bacterial to the fungi was studied. The results (Figure 1A) showed that *P. aeruginosa* PAO1 adhesion to living LG-8 cells occurred at the tested pH values (2.0–9.0), and is prominent in weakly acidic and alkaline environments. It showed high efficiency of the pathogen adhesion to the living fungi LG-8, from 40.4% to 58.1%, in acidic environments with pH values ranging from 2.0 to 6.0. It is potential that LG-8 cells can be used to treat stomach inflammation and in acidic food due to its acid tolerance. On the other hand, the efficiency of the pathogen adhesion to the living fungi LG-8 at pH 8.0 and pH 9.0 was 56.3% and 48.2%, respectively, staying high in a weakly alkaline environment. Compared to the results obtained by Tiago et al., LG-8 showed great tolerance to a wide range of pH values [23].

Figure 1C showed that the efficiency of PAO1 adhesion to dead LG-8 cells was 32.7–75% in pH 2 to 9 conditions. The dead fungus showed similar adhesion efficiency to the living fungus in the acidic solutions (pH 2–6). However, the dead fungus showed higher adhesion efficiency than the living ones in the alkaline solutions (pH 7–9). Therefore, the dead LG-8 has an advantage over the living ones to adhere PAO1 in a wide pH range.

### 3.3. Effect of Oxgall Bile Salt on P. aeruginosa PAO1 adhesion

Bile secreted by the gallbladder generally causes the physiological concentration of intestinal bile salts to vary from 0.2% to 2% [24,25]. Resistance to bile salt is thereby necessary for LG-8 to be applied as a probiotic. Therefore, the influence of oxgall bile salts on adhesion of *P. aeruginosa* PAO1 to living (Figure 1B) and sterilized (Figure 1D) LG-8 was studied. The adhesion efficiency (Figure 1B) increased from 21.9% to 45.1% as oxgall bile salt concentrations increased from 0.1% to 0.3%, plummeted to 11.7% at 0.5% oxgall bile salts, and then continued to drop to 7.6% at 1% oxgall bile salts. The present investigation shows that *P. aeruginosa* PAO1 can adhere to living LG-8 at oxgall bile salt concentrations ranging from 0.1% to 1%. On the other hand, the maximum efficiency of PAO1 adhesion to LG-8 was 83.48% in 0.5% bile salt. Although the sterilized LG-8 could trap much more PAO1 than the living ones in the bile salt (0.1% to 2%), the sterilized and the living LG-8 showed similar up-down trend of adhesion efficiency.

### 3.4. Visualization by Optical Microscopy

**Figure 2:**
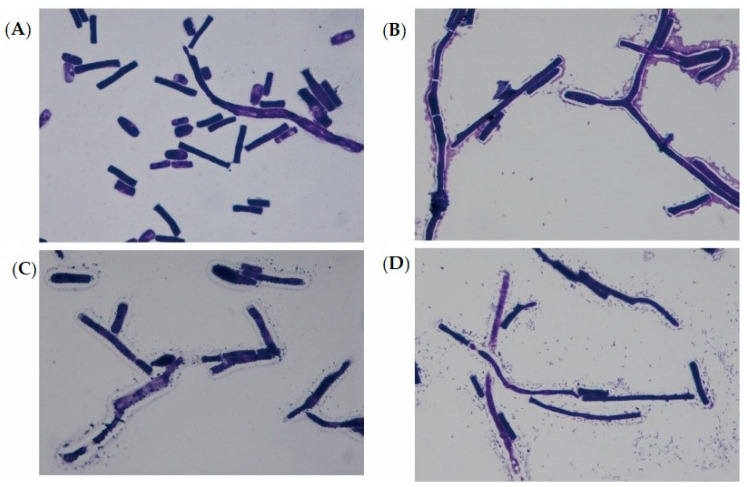
Visualization by optical microscopy. (**A**,**B**): The unsterilized *G. candidum* LG-8 cells untreated with (**A**) and treated (**B**) with *P. aeruginosa* PAO1 visualized by optical electron microscopy; (**C**,**D**): The sterilized *G. candidum* LG-8 cells untreated with (**C**) and treated (**D**) with *P. aeruginosa* PAO1.

The morphology of LG-8 cells and occurrence of PAO1 adhesion to the fungus can be visualized quickly and directly by optical microscopy. Figure 2 shows unsterilized LG-8 before (**A**) and after (**B**) exposure to *P. aeruginosa* PAO1. Figure 2A shows that LG-8 cells before exposure to *P. aeruginosa* PAO1 were rod/drum shaped and uneven in size. Figure 2B shows that several layers of *P. aeruginosa* PAO1 adhered to surface of LG-8 cells, indicating the significant ability of unsterilized LG-8 trapping the PAO1. Furthermore, white capsular wall surrounding the LG-8 cells presented. However, the capsular walls of sterilized LG-8 cells were blurred (Figure 2C), and the decreased *P. aeruginosa* PAO1nwere trapped in a diffuse cell surface of sterilized LG-8 (Figure 2D). Optical microscopy provides a way for us to quickly visualize the adhesion of *P. aeruginosa* PAO1 to LG-8 cell wall.

### 3.5. Analysis by LSCM

Fluorescein isothiocyanate labeled Concanavalin A (FITC-ConA) can bind a variety of polysaccharides, especially those in biofilms, and emit green fluorescence [26]. PI can enter nonviable cells to combine with DNA (Deoxyribonucleic acid) and then emit red fluorescence [27]. Therefore, LSCM was applied to further confirm that *P. aeruginosa* PAO1 can adhere to dead LG-8 cells and to determine whether the mechanism involved extracellular polysaccharides of the fungus.

**Figure 3:**
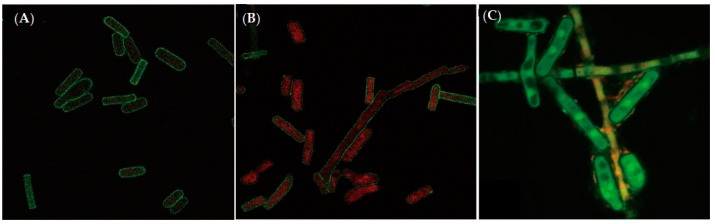
Visualization by laser scanning confocal microscopy (LSCM). (**A**): micrographs of *Geotrichum candidum* LG-8 untreated with *P. aeruginosa* PAO1 before (**A**) and after (**B**) the pathogen adhesion assays; (**C**): The *G. candidum* LG-8 treated with *P. aeruginosa* PAO1.

Figure 3A,B represents the *G. candidum* LG-8 cells untreated with *P. aeruginosa* PAO1. The presence of highly dark *G. candidum* LG-8 cells (Figure 3A) with weak green-fluorescent edges before incubation with bacterial cells, revealed that insufficient polysaccharides existed in cell walls. A few red cells (Figure 3B) surrounded by week green fluorescence represented dead LG-8 cells, indicating low survival of LG-8 cells with intact membranes after incubation for 4 h and no significant increase in polysaccharide. Figure 3C shows LSCM micrographs of *G. candidum* LG-8 cells treated with *P. aeruginosa* PAO1. The presence of green LG-8 cells indicated that the viable LG-8 cells can trap PAO1.

Additionally, some green fungal cells with large orange spots (Figure 3C) were observed, which were reported to be compromised cells by Kuyukina et al. [20,28]. Adhesion of red, green, and orange bacterial cells to the compromised fungal cells indicated that dead, living, and compromised bacterial cells were adhered to the compromised cells. The visible surfaces and edges of LG-8 cells treated with PAO1 (Figure 3C) emitted bright green fluorescence, while the control group (Figure 3A,B) showed weak green-fluorescent edges, indicating that the polysaccharide on the surface of LG-8 increased after treatment with *P. aeruginosa* PAO1.

### 3.6. Analysis by SEM

**Figure 4:**
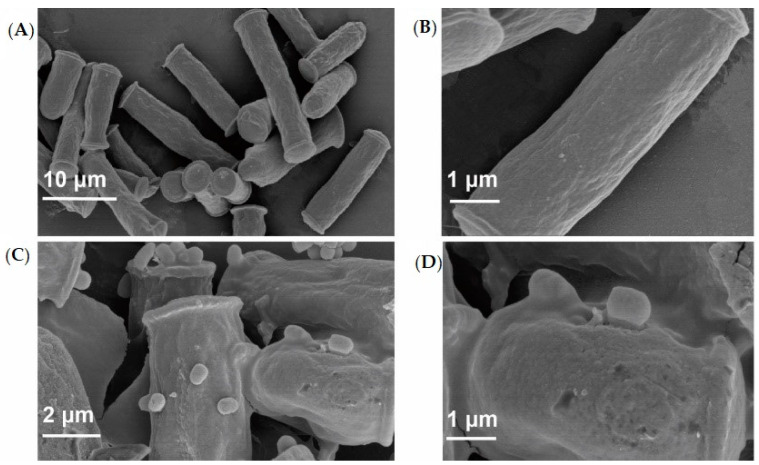
Visualization by scanning electron microscopy (SEM). (**A**,**B**): SEM micrographs of *G. candidum* LG-8 untreated (**A**) and treated (**C**) with *P. aeruginosa* PAO1; (**B**,**D**): (**B**,**D**) were micrographs of (**A**,**C**) at magnification, respectively.

SEM images were obtained to show the morphological characteristics of cells visually and evaluate the adhesion between *P. aeruginosa* PAO1 and *G. candidum* LG-8 cells via magnified visualization. As shown in Figure 4A,B, the LG-8 cells untreated with *P. aeruginosa* PAO1 were structurally intact, plump, and drum shaped. The edges were neat, while the surfaces were smooth. After treatment with *P. aeruginosa* PAO1 (Figure 4C,D), small holes appeared on the fungal surfaces. Furthermore, the fungal edges, especially the sites of trapping the bacteria, were irregular. The envelope-like edge (Figure 4D) is associated with the capsule (Figure 1B) visualized by a normal microscope in this study. Regardless of adhesion occurring among LG-8 cells or between LG-8 cells and the bacteria, LG-8 cells were coated in a syrup-like substance. The syrup-like EPS was speculated to be polysaccharide, which was also visualized by LSCM analysis in this study.

### 3.7. Analysis by TEM

TEM is usually applied to investigate bacterial adhesion, because it was previously proved to provide high-resolution images of structures associated with adhesion between bacteria and other organisms [29]. In addition, both LSCM and SEM analysis showed that polysaccharide on the surface of *G. candidum* LG-8 played a vital role in adhering *P. aeruginosa* PAO1. TEM was thereby applied to show whether the structure, especially the cell wall, of LG-8 cells contribute to adhere *P. aeruginosa* PAO1 at the ultrastructural level.

**Figure 5:**
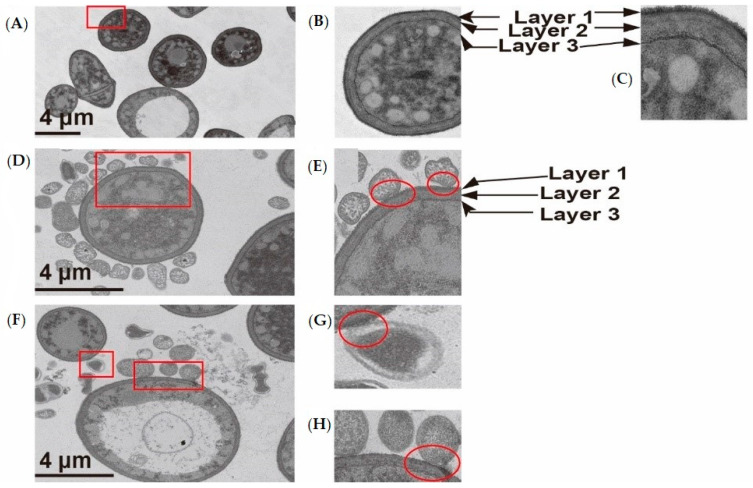
Visualization by transmission electron microscopy (TEM). (**A**,**B**): TEM micrographs of *G. candidum* LG-8 untreated with *P. aeruginosa* PAO1; (**D**–**H**): TEM micrographs of *G. candidum* LG-8 treated with *P. aeruginosa* PAO1; Notes: (**B**,**C**,**E**,**G**,**H**) were obtained from the red boxed fields of (**A**,**D**,**F**) at magnification, respectively.

The extracellular structure of LG-8 cell untreated with the bacteria (Figure 5A–C) was composed of three layers, which were proved to be mannose (layer 1), glucan (layer 2), and chitin (layer 3) by Hudson et al. [13]. After being treated with *P. aeruginosa* PAO1, the extracellular structure of LG-8 (Figure 5D,E) still consisted of three layers. Previous reports by Ganner et al. [30] demonstrated that yeast probiotics and their EPS can adhere to enteropathogenic bacteria, thereby reducing the adhesion ability of enteric pathogens to host cells [30]

Although there was an obvious ring of bacteria around LG-8 (Figure 5D), not all bacteria and LG-8 were directly linked due to centrifugation and the reagents. It is thus difficult to accurately determine the thickness of the surface EPS (especially the outermost carbohydrate layer) by TEM. However, we observed irregular intracytoplasmic structures with variable electron density in the LG-8 cells (Figure 5D,E). A pedestal-like structure with a high electron density was formed on the surfaces of some viable bacteria during the adhesion process (Figure 5E, red ellipse), which indicated that trapping of living bacteria by fungi was associated with active behavior of the bacteria. Thus, further studies will be required. In Figure 5F, the dead LG-8 still trapped PAO1 cells, which were magnified to visualize the adhesion in Figure 5G. Additionally, the dead PAO1 cells were bound to the LG-8 surface with a magnified visualization in Figure 5H. The results observed by TEM were consistent with that obtained by SEM and LSCM.

### 3.8. Analysis by AFM

In recent years, AFM has been applied to visualize and quantify the forces that guide bacteria onto the surfaces of materials [31,32]. Centrifugation and reagents are required for sample preparation before TEM analysis and SEM analysis. Consequently, the cell morphology and the original adhesion were possibly changed. AFM is thereby recommended for observing the adhesion of *P. aeruginosa* PAO1 to LG-8 because of the simple sample preparation in absence of reagents. Besides, it is unlikely that aggregation driven by evaporation would preferentially result in adhesion through edge sites. In addition to LSCM, SEM, and TEM, AFM was thereby expected to be a complementary technology for surface characterization of fungi associated with bacterial adhesion in our study.

**Figure 6:**
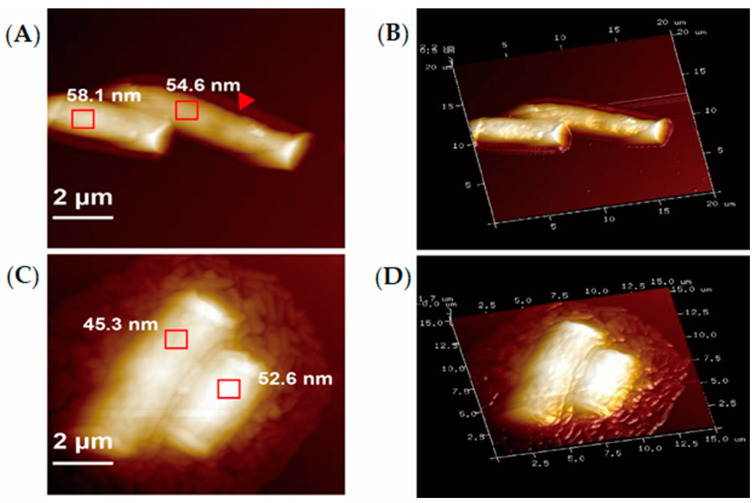
Visualization by atomic force microscopy (AFM). (**A**,**C**): AFM micrographs of unsterilized *G. candidum* LG-8 untreated (**A**) and treated (**B**) with *P. aeruginosa* PAO1. (**C**,**D**): (**B**,**D**) represent the three-dimensional micrographs of (**A**,**B**), respectively.

For unsterilized *G. candidum* LG-8 (Figure 6A,B) untreated with *P. aeruginosa* PAO1, the morphological characteristics were consistent with the results by SEM. The flattened structures surrounding the LG-8 cells observed by optical microscopy and SEM were also observed by AFM (Figure 6A, red arrow). It has been previously reported that the flattened structure is the cell wall undergoing the dry process [33]. Our high-resolution AFM images (Figure 6C,D) provided compelling evidence of bacterial cell adhesion onto the surfaces of the fungus LG-8. The morphology of the fungi (Figure 6C,D) after treatment with PAO1 did not change significantly. However, the Ra of selected areas in *G. candidum* LG-8 cells decreased from 54.6 and 58.1 nm (Figure 6A, red rectangle) to 45.3 and 52.6 nm Figure 6B, red rectangle) after treatment with the bacteria.

## 4. Discussion

The adhesion of *P. aeruginosa* PAO1 to the fungal LG-8 occurred in a very short time (1 h), indicating that adhesion of PAO1 to the surfaces of LG-8 cells was a mechanism of trapping PAO1 by LG-8 cells. Approximately one hundred-fold of *P. aeruginosa* PAO1 were removed by both unsterilized and sterilized LG-8 biomass, indicating the strong ability of LG-8 to trap the bacterium comparing with results obtained by Tiago et al. [23]. The sterilized and unsterilized LG-8 cells showed similar capacity to trap the PAO1. The similar results indicated that the possibility of applying sterilized fungal LG-8 cells to treat immunocompromised patients, in which case the use of living strains is not proposed due to the possible occurrence of opportunistic infections [34]. Additionally, this method avoids other biological problems caused by the viability of the fungus, such as reproduction in the environment and modification of food quality. Compared to the results obtained by Tiago et al., LG-8 showed great tolerance to a wide range of pH values [23]. The pH-tolerance fungus is a candidate for pathogen bio-adsorbent in water, food, and human body.

The up-down trend of adhesion efficiency in different oxgall bile salt solution showed that PAO1 adhesion occurred at low oxgall bile salt levels, but was inhibited at high concentrations of oxgall bile salts. This result is different from those of previous studies by Tiago et al., which demonstrated that adhesion efficiency was not obvious when bile salts were added after yeast growth [23]. Moreover, the paper reported that a high concentration (0.3%) of bile salts inhibited adhesion [23]. A certain report showed that pre-exposure of probiotics to a low level of bile salts can enhance their ability to tolerate high concentrations of bile [35]. Another report indicated that the bacteria M92 with rough shape were more sensitive to bile salt comparing to the smooth cells, because the rough cells have a more compact longer-chain structure [36]. Therefore, the probiotics tolerance to bile salt is dependent on species, and possibly influenced by pH, viability of the strain, and other factors. Consequently, it is hard to clarify the real mechanism involved in probiotics tolerance to bile salt.

The capsular wall surrounding the unsterilized LG-8 cells interacted with the bacterium, indicating that the EPS of the fungi increased for adhesion. The capsular wall surrounding the sterilized LG-8 cells after bacterium-exposure were destroyed by sterilization. The change of the capsular wall revealed the correlation of EPS and PAO1 adhesion. A number of investigations showed EPS on the cell wall of the fungi play a vital role in functional activities [37,38]. Surface of yeast were reported to contain proteins and polysaccharides, which generally were the provider of functional groups [13].

By LSCM, adhesion of PAO1 cells to surface of the living and compromised fungal cells showing PAO1 binding by the initiative LG-8 was in part a mechanism. The increased polysaccharide on the surface of LG-8 after treatment with *P. aeruginosa* PAO1 indicated that polysaccharides of the fungal cells participated in the adhesion of *P. aeruginosa* PAO1 to *G. candidum* LG-8.

By SEM, the coverage of PAO1 related to the syrup-like EPS on LG-8 surface, at least showing that the adhesion and EPS was mechanisms of bacterial adhesion to LG-8 cells. The trapping of bacteria on material surfaces is a complex biological process affected by numerous parameters, such as surface charge, material composition, and surface topography [33,39]. On the other hand, EPS including polysaccharides, can also influence the adhesion of bacteria to LG-8 in many ways [40,41]. Strains are rarely used to remove pathogenic bacteria because of specificity. Tiago et al. also used fungal cells to capture pathogenic bacteria [24]. However, no previous studies have examined the EPS-mediated *P. aeruginosa* adhesion to *Geotrichum*.

By TEM, the PAO1 ring around LG-8 cells showed the PAO1 adhesion to the LG-8 surface. However, the PAO1 cells were not attached to the LG-8 cells because of the absence of EPS between the LG-8 and PAO1. The disappearance of EPS is possibly due to the solvents used for sample pretreatment before the TEM visualization. Therefore, it is not suitable to justify the effect of EPS on adhesion of PAO1 to LG-8 by measuring the thickness of the LG-8 cell wall.

By AFM, the flattened structures surrounding the cells, the structure was reported to be due to the drying process and/or structure of the cell wall [33]. These changes in roughness indicated that the nano-scale roughness of the fungal LG-8 surface played a role in assisting the adhesion process. The rough surfaces, like the wrinkle intestinal wall, provided a larger area for the bacteria to adhere to the fungi LG-8.

## 5. Conclusions

*G. candidum* LG-8 efficiently traps *P. aeruginosa* PAO1 over wide ranges of oxgall bile salt concentrations and pH values. It indicated that *G. candidum* LG-8 is a potential probiotic candidate. Multiple analyses by microscopy were performed to reveal the mechanism and provide an investigation model. Optical microscope, SEM, and AFM showed PAO1 binding by the initiative LG-8 and polysaccharide of LG-8 was in part mechanisms of PAO 1 adhesion to LG-8. Other investigations via multi-scale methods showed fungal polysaccharides and a pedestal-like bacterial structure contributed to the adhesion of *P. aeruginosa* PAO1 to *G. candidum* LG-8. Moreover, roughness of LG-8 changed in the adhesion process. Additional studies will be required to further understand these adhesion mechanisms. The study provides a theory basis for applying dead fungal *G. candidum* LG-8 to treat *P. aeruginosa* PAO1 contamination in water, food, and infection in the immunocompromised human body.

## Figures and Tables

**Table 1:** Influence of time and LG-8 viability on adhesion of *P. aeruginosa* PAO1 to *G. candidum* LG-8.

Carbohydrate	Observing Time (h)/Strain Counts [log CFU/mL]		
	1 h	2 h	4 h
Control group	+	+	+/7.01 ± 0.01a
sterilized strains	+	+	+/5.13 ± 0.14b
unsterilized strains	+	+	+/4.96 ± 0.25c

Notes: Observed in different hours was expressed as +. Values were the mean ± standard deviations. Values with different small letters are significantly different at HSD (*p* < 0.05).

## Abbreviations

EPS: Extracellular polymeric substances. CFU: Colony-forming units; PI: Propidium iodide; FITC-ConA: Fluorescein isothiocyanate labeled Concanavalin A; LSCM: Laser scanning confocal microscope; SEM: Scanning electron microscope; TEM: Transmission electron microscope; AFM: Atomic force microscope.

## References

[1] SamaržIja D., Zamberlin Š., Pogačic T. (2012). Psychrotrophic bacteria and their negative effects on milk and dairy products quality. Mljekarstvo Cas. Unaprjeđenje Proizv. Prerade Mlijeka.

[2] Wang Y.M., Tang Z., Qiao X., Qin S., Ni Y.L., Zheng D.Y. (2015). Survey of Pseudomonas aeruginosa contamination in barreled water in Jiangsu province. Chin. J. Health Lab. Technol..

[3] Wang C., Zhang J.J., Yang X.R., Tang Y.J., Cui X.W., Huang Y. (2019). Investigation of Pseudomonas aeruginosa contamination in barreled drinking water in Sichuan province and study on its prevention and control measures. J. Food Saf. Qual..

[4] Malhotra S., Hayes D., Wozniak D.J. (2019). Mucoid Pseudomonas aeruginosa and regional inflammation in the cystic fibrosis lung. J. Cyst. Fibros..

[5] He J., Jia X., Yang S., Xu X., Sun K., Li C., Yang T., Zhang L. (2018). Heteroresistance to carbapenems in invasive Pseudomonas aeruginosa infections. Int. J. Antimicrob. Agents.

[6] Kipnis E., Sawa T., Wiener-Kronish J. (2006). Targeting mechanisms of Pseudomonas aeruginosapathogenesis. Méd. Mal. Infect..

[7] Ofori I., Maddila S., Johnson L., Jonnalagadda S.B. (2018). Chlorine dioxide inactivation of Pseudomonas aeruginosa and Staphylococcus aureus in water: The kinetics and mechanism. J. Water Process. Eng..

[8] Tsao L.-H., Hsin C.-Y., Liu H.-Y., Chuang H.-C., Chen L.-Y., Lee Y.-J. (2018). Risk factors for healthcare-associated infection caused by carbapenem-resistant Pseudomonas aeruginosa. J. Microbiol. Immunol. Infect..

[9] Trapnell B.C., Mccolley S.A., Kissner D.G., Rolfe M.W., Rosen J.M., Matthew M.K., Lisa M., Bruce M., Geller D.E. (2012). Fosfomycin/tobramyscin for inhalation in patients with cystic fibrosis with pseudomonas airway infection. Am. J. Respir. Crit. Care Med..

[10] Fang J., Liu H., Shang C., Zeng M., Ni M., Liu W.E. (2013). coli and bacteriophage MS2 disinfection by UV, ozone and the combined UV and ozone processes. Front. Environ. Sci. Eng..

[11] Li J., Ding T., Liao X., Chen S., Ye X., Liu D. (2017). Synergetic effects of ultrasound and slightly acidic electrolyzed water against Staphylococcus aureus evaluated by flow cytometry and electron microscopy. Ultrason. Sonochem..

[12] Tashiro Y., Yawata Y., Toyofuku M., Uchiyama H., Nomura N. (2013). Interspecies Interaction between Pseudomonas aeruginosa and Other Microorganisms. Microbes Environ..

[13] Hudson L.E., McDermott C.D., Stewart T.P., Hudson W., Rios D., Fasken M.B., Corbett A.H., Lamb T.J. (2016). Characterization of the Probiotic Yeast Saccharomyces boulardii in the Healthy Mucosal Immune System. PLoS ONE.

[14] Rodriguez-Nogales A., Algieri F., Garrido-Mesa J., Vezza T., Utrilla M.P., Chueca N., García F., Rodríguez-Cabezas M.E., Gálvez J. (2018). Intestinal anti-inflammatory effect of the probiotic Saccharomyces boulardii in DSS-induced colitis in mice: Impact on microRNAs expression and gut microbiota composition. J. Nutr. Biochem..

[15] Grilli D.J., Mansilla M.E., Giménez M.C., Sohaefer N., Ruiz M.S., Terebiznik M.R., Sosa M., Arenas G.N. (2019). Pseudobutyrivibrio xylanivorans adhesion to epithelial cells. Anaerobe.

[16] Virji M. (2008). Ins and Outs of Microbial Adhesion. Top. Curr. Chem..

[17] Hamedi H., Misaghi A., Modarressi M.H., Salehi T.Z., Khorasanizadeh D., Khalaj V. (2013). Generation of a Uracil Auxotroph Strain of the Probiotic Yeast Saccharomyces boulardii as a Host for the Recombinant Protein Production. Avicenna J. Med Biotechnol..

[18] Li J., Ahn J., Liu N., Chen S., Ye X., Ding T. (2016). Evaluation of Ultrasound-Induced Damage to Escherichia coli and Staphylococcus aureus by Flow Cytometry and Transmission Electron Microscopy. Appl. Environ. Microbiol..

[19] Li J., Suo Y., Liao X., Ahn J., Liu D., Chen S., Ye X., Ding T. (2017). Analysis of Staphylococcus aureus cell viability, sublethal injury and death induced by synergistic combination of ultrasound and mild heat. Ultrason. Sonochem..

[20] Kuyukina M.S., Ivshina I.B., Korshunova I.O., Rubtsova E.V. (2014). Assessment of bacterial resistance to organic solvents using a combined confocal laser scanning and atomic force microscopy (CLSM/AFM). J. Microbiol. Methods.

[21] Vandevoorde L., Christiaens H., Verstraete W. (1992). Prevalence of coaggregation reactions among chicken lactobacilli. J. Appl. Bacteriol..

[22] Chen S.H., Cheow Y.L., Ng S.L., Ting A.S.Y. (2019). Mechanisms for metal removal established via electron microscopy and spectroscopy: A case study on metal tolerant fungi Penicillium simplicissimum. J. Hazard. Mater..

[23] Tiago F.C.P., Martins F.S., Souza E.L.S., Pimenta P.F.P., Araújo H.R.C., Castro I.M., Brandao R.L., Nicoli J.R. (2012). Adhesion to the yeast cell surface as a mechanism for trapping pathogenic bacteria by Saccharomyces probiotics. J. Med Microbiol..

[24] Prouty A.M., Schwesinger W.H., Gunn J.S. (2002). Biofilm Formation and Interaction with the Surfaces of Gallstones by Salmonella spp.. Infect. Immun..

[25] Prouty A.M., Van-Velkinburgh J.C., Gunn J.S. (2002). Salmonella enterica serovar typhimurium resistance to bile: Identification and characterization of the lQRA cluster. J. Bacteriol..

[26] Kalia M., Yadav V.K., Singh P.K., Sharma D., Narvi S.S., Agarwal V. (2018). Exploring the impact of parthenolide as anti-quorum sensing and anti-biofilm agent against Pseudomonas aeruginosa. Life Sci..

[27] Ripolles-Avila C., Hascoët A., Guerrero-Navarro A., Rodríguez-Jerez J. (2018). Establishment of incubation conditions to optimize the in vitro formation of mature Listeria monocytogenes biofilms on food-contact surfaces. Food Control.

[28] Roberts A.E.L., Maddocks S.E., Cooper R. (2012). Manuka honey is bactericidal against Pseudomonas aeruginosa and results in differential expression of oprF and algD. Microbiology.

[29] Grin I., Schwarz H., Linke D. (2011). Electron Microscopy Techniques to Study Bacterial Adhesion. Adv. Exp. Med. Biol..

[30] Ganner A., Stoiber C., Wieder D., Schatzmayr G. (2010). Quantitative in vitro assay to evaluate the capability of yeast cell wall fractions from Trichosporon mycotoxinivorans to selectively bind gram negative pathogens. J. Microbiol. Methods.

[31] Wu S., Altenried S., Zogg A., Zuber F., Maniura-Weber K., Van Der Mei H.C. (2018). Role of the Surface Nanoscale Roughness of Stainless Steel on Bacterial Adhesion and Microcolony Formation. Am. Chem. Soc. Omega.

[32] Musashi T., Yusuke M., Jun I., Chiaki O., Akihiko K. (2015). The mapping of yeast’s G-protein coupled receptor with an atomic force microscope. Nanoscale.

[33] Huang Q., Wu H., Cai P., Fein J.B., Chen W. (2015). Atomic force microscopy measurements of bacterial adhesion and biofilm formation onto clay-sized particles. Sci. Rep..

[34] Boyle R.J., Robins-Browne R.M., Tang M.L.K. (2006). Probiotic use in clinical practice: What are the risks?. Am. J. Clin. Nutr..

[35] Begley M., Gahan C.G., Hill C. (2005). The interaction between bacteria and bile. FEMS Microbiol. Rev..

[36] Šušković J., Kos B., Matošić S., Besendorfer V. (2000). The effect of bile salts on survival and morphology of a potential probiotic strain Lactobacillus acidophilus M92. World J. Microbiol. Biotechnol..

[37] Jeong D., Kim D., Kang I.-B., Kim H., Song K.-Y., Kim H.-S., Seo K.-H. (2017). Characterization and antibacterial activity of a novel exopolysaccharide produced by Lactobacillus kefiranofaciens DN1 isolated from kefir. Food Control.

[38] Ben Taheur F., Fedhila K., Chaieb K., Kouidhi B., Bakhrouf A., Abrunhosa L. (2017). Adsorption of aflatoxin B1, zearalenone and ochratoxin A by microorganisms isolated from Kefir grains. Int. J. Food Microbiol..

[39] Palmer J., Flint S., Brooks J. (2007). Bacterial cell attachment, the beginning of a biofilm. J. Ind. Microbiol. Biotechnol..

[40] Levy D.E., Tang P.C., Musser J.H. (1994). Chapter 22. Cell Adhesion and Carbohydrates. Ann. Rep. Med. Chem..

[41] Taverniti V., Via A.D., Minuzzo M., Del Bo’ C., Riso P., Frøkiær H., Guglielmetti S. (2017). In vitro assessment of the ability of probiotics, blueberry and food carbohydrates to prevent S. pyogenes adhesion on pharyngeal epithelium and modulate immune responses. Food Funct..

